# Randomised sham-controlled trial of transcutaneous electrical stimulation in obstructive sleep apnoea

**DOI:** 10.1136/thoraxjnl-2016-208691

**Published:** 2016-07-19

**Authors:** Martino F Pengo, Sichang Xiao, Culadeeban Ratneswaran, Kate Reed, Nimish Shah, Tao Chen, Abdel Douiri, Nicholas Hart, Yuanming Luo, Gerrard F Rafferty, Gian Paolo Rossi, Adrian Williams, Michael I Polkey, John Moxham, Joerg Steier

**Affiliations:** 1 Faculty of Life Sciences and Medicine, King's College London, London, UK; 2 Guy's and St Thomas’ NHS Foundation Trust, Lane Fox Respiratory Unit/Sleep Disorders Centre, London, UK; 3 Department of Medicine (DIMED), University of Padua, Padua, Italy; 4 State Key Laboratory of Respiratory Disease, The First Affiliated Hospital of Guangzhou Medical University, Guangzhou, China; 5 Division of Health and Social Care, King's College London, London, UK; 6 NIHR Respiratory Biomedical Research Unit, The Royal Brompton and Harefield NHS Foundation Trust and Imperial College, London, UK

**Keywords:** Sleep apnoea

## Abstract

**Introduction:**

Obstructive sleep apnoea (OSA) is characterised by a loss of neuromuscular tone of the upper airway dilator muscles while asleep. This study investigated the effectiveness of transcutaneous electrical stimulation in patients with OSA.

**Patients and methods:**

This was a randomised, sham-controlled crossover trial using transcutaneous electrical stimulation of the upper airway dilator muscles in patients with confirmed OSA. Patients were randomly assigned to one night of sham stimulation and one night of active treatment. The primary outcome was the 4% oxygen desaturation index, responders were defined as patients with a reduction >25% in the oxygen desaturation index when compared with sham stimulation and/or with an index <5/hour in the active treatment night.

**Results:**

In 36 patients (age mean 50.8 (SD 11.2) years, male/female 30/6, body mass index median 29.6 (IQR 26.9–34.9) kg/m^2^, Epworth Sleepiness Scale 10.5 (4.6) points, oxygen desaturation index median 25.7 (16.0–49.1)/hour, apnoea-hypopnoea index median 28.1 (19.0–57.0)/hour) the primary outcome measure improved when comparing sham stimulation (median 26.9 (17.5–39.5)/hour) with active treatment (median 19.5 (11.6–40.0)/hour; p=0.026), a modest reduction of the mean by 4.1 (95% CI −0.6 to 8.9)/hour. Secondary outcome parameters of patients' perception indicated that stimulation was well tolerated. Responders (47.2%) were predominantly from the mild-to-moderate OSA category. In this subgroup, the oxygen desaturation index was reduced by 10.0 (95% CI 3.9 to 16.0)/hour (p<0.001) and the apnoea-hypopnoea index was reduced by 9.1 (95% CI 2.0 to 16.2)/hour (p=0.004).

**Conclusion:**

Transcutaneous electrical stimulation of the pharyngeal dilators during a single night in patients with OSA improves upper airway obstruction and is well tolerated.

**Trial registration number:**

NCT01661712.

**Key messages**

**What is the key question?**

- Can electrical current be delivered non-invasively via transcutaneous patches with ongoing stimulation for the entire night?

**What is the bottom line?**

- The current trial provides evidence that transcutaneous electrical stimulation of the upper airway dilator muscles, a non-invasive approach, delivered throughout the night improves sleep apnoea.

**Why read on?**

- It is important to further refine transcutaneous electrical stimulation of the upper airway dilator muscles, as this method offers a promising and novel approach in the range of non-CPAP therapies for patients with obstructive sleep apnoea.

## Introduction

Obstructive sleep apnoea (OSA) is the most common form of sleep-disordered breathing^1^ and is widely recognised as a risk factor for cardiovascular diseases.^2^ The prevalence of OSA continues to rise, imposing a worldwide burden on public health and currently affecting 10% of middle-aged men and 3% of women aged 30–49 years in the USA.^3^ The principle evidence-based treatment for OSA, in addition to weight loss, is continuous positive airway pressure (CPAP);^4^ however, CPAP is frequently not tolerated over longer periods, with a quarter of patients being non-compliant within weeks and half of all patients not using the equipment after 1 year.^5^ Alternative therapies are needed to reduce symptoms and health risks for patients who fail CPAP treatment.

In 1978, Remmers *et al*^6^ described the pathophysiology of upper airway obstruction in sleep apnoea. Since then several research groups have observed that electrical stimulation of the upper airway could result in an increased tone of the dilator muscles of the upper airway,^7^ thereby enabling patients to maintain a patent upper airway while asleep.^8^ ^9^ In 2014, hypoglossal nerve stimulation using an implantable stimulator was approved by the US Food and Drug Administration for the treatment of OSA following earlier publication of the results of the STAR trial.^10^

Previously, Miki *et al*^11^ ^12^ demonstrated that transcutaneous electrical stimulation was effective in reducing the apnoea index and duration and improved oxygen saturation; however, other researchers could not replicate these results.^13^ ^14^ In 2011, our group showed that continuous transcutaneous electrical stimulation (CTES) was a feasible and effective approach to stimulate the upper airway dilator muscles during short periods while asleep.^15^

In this trial, the aim was to conduct a randomised, sham-controlled and double-blind clinical trial to test the effectiveness and safety of overnight transcutaneous electrical stimulation of the upper airway muscles in patients with OSA.

## Patients and methods

**Figure 1:**
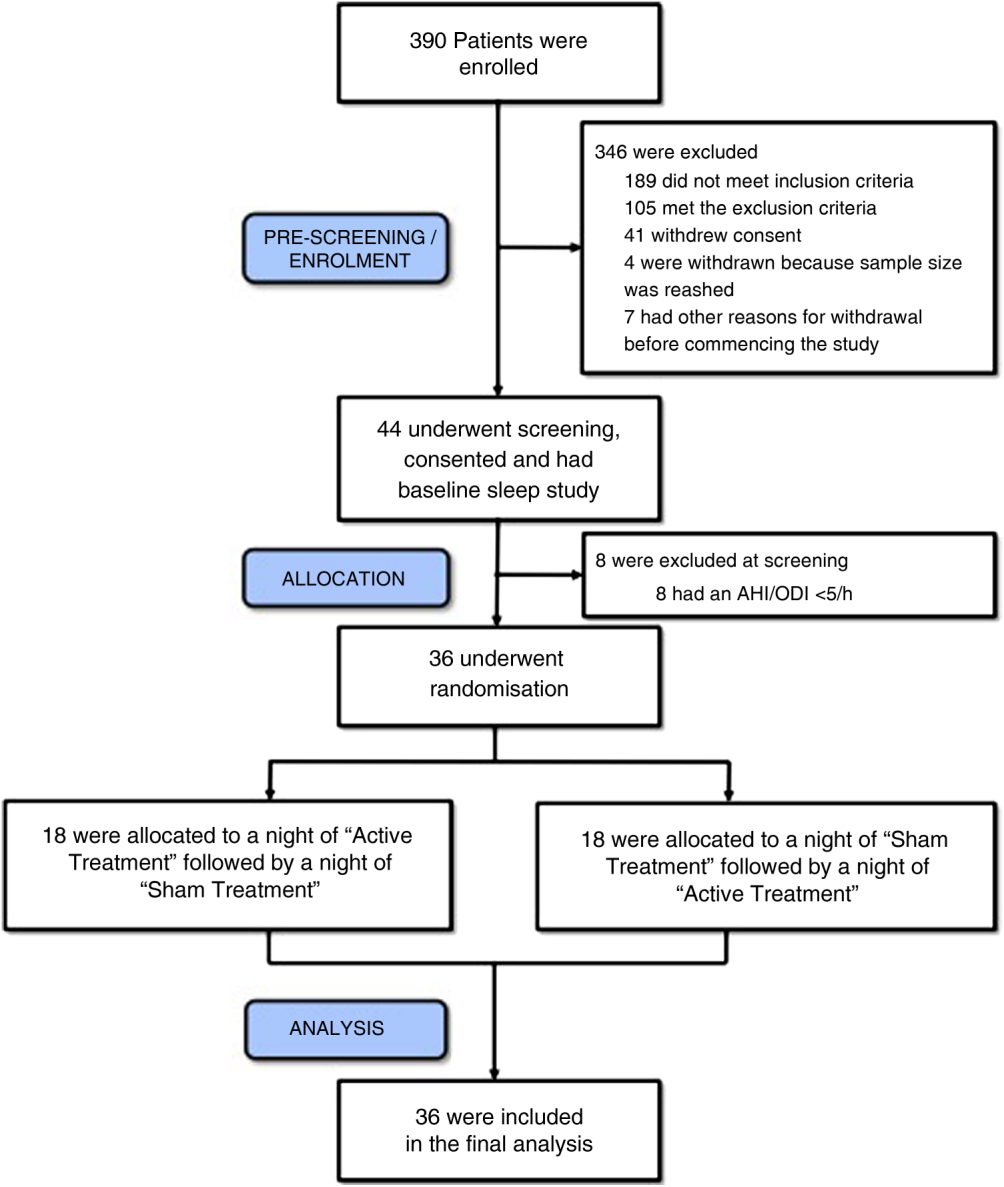
Consort diagram for the TESLA trial. AHI, apnoea-hypopnoea index; ODI, oxygen desaturation index.

This sham-controlled and randomised crossover trial was approved by the London ethics committee for clinical trials (London-Dulwich, UK; 12/LO/1428) and was registered in ClinicalTrials.gov (NCT01661712). We enrolled patients referred to Guy’s and St Thomas’ and the Royal Brompton & Harefield NHS Foundation Trusts sleep services (both London, UK) for treatment of OSA between March 2013 and December 2015 when we achieved the full sample size. All patients were provided with a patient information sheet and informed written consent was obtained prior to enrolment (figure 1).^16^

### Inclusion and exclusion criteria

The study included patients aged 18–75 years with a body mass index (BMI) between 18 and 40 kg/m^2^ who had OSA with an oxygen desaturation index (4%ODI) ≥15/hour or with an ODI ≥5/hour plus an Epworth Sleepiness Scale (ESS) >10 points. Exclusion criteria were obesity-hypoventilation syndrome, significant airway obstruction, acute or critical illness.

### Polysomnography

Patients underwent nocturnal full polysomnography (Alice5 equipment, Respironics, Murrysville, Pennsylvania, USA) at baseline and during randomly allocated sham electrical stimulation and active electrical stimulation nights. These three nights were recorded with a gap of at least 3 days to provide ‘wash-out’ periods.

### Interventional sleep studies

Patients were randomised in a crossover design into one of two treatment arms; either they underwent polysomnography plus CTES during the first study night (‘active treatment’) followed by another polysomnography plus sham-CTES during a second night, at least three nights later (‘washout period’) or they underwent the tests in the reverse order (figure 1).

### Transcutaneous electrical stimulation

To deliver CTES, a specifically tailored electrical stimulator was employed (SOSATS device, Morgan Innovation and Technology/MIAT, Petersfield, UK) which was connected to a laptop (Toshiba, Tokyo/Japan) via a standard USB cable. A stimulation lead from the device was attached bilaterally to the patient's neck via two 4×4 cm patches (Verity Medical, Hampshire, UK) placed bilaterally halfway between the chin and the angle of the mandible over the submental area, as previously described (see online supplementary material).^15^ Sham-CTES included the placement of the stimulation patches and a stimulation marker that was displayed on the computer screen; no electrical current was applied.

10.1136/thoraxjnl-2016-208691.supp1 Supplementary material

### Outcome measures

The primary outcome measure for this trial was the 4%ODI/hour of sleep (events/hour). The 4%ODI was chosen as the primary outcome parameter in preference to the apnoea-hypopnoea index (AHI). Secondary outcome measures were AHI, nocturnal oxygen saturation levels and nadir oxygenation and sleepiness, as measured by the Stanford Sleepiness Scale. Patient comfort and device acceptance were measured by administering ad-hoc visual analogue scales on waking after sham and stimulation sleep studies (see online supplementary material). Last, a post-hoc analysis was performed to determine responders whose ODI had improved by >25% compared with sham night and/or the total ODI was <5/hour.

### Sample size calculation

A sample size calculation for the primary outcome, 4%ODI, was performed based on the results of a feasibility study.^15^ A sample size of 30 patients would achieve 90% power to detect a mean of paired differences between the treatments of 17.9 events/hour with an estimated SD of differences of 18.9 events/hour and with a significance level of 5% using a two-sided Wilcoxon test assuming that the actual distribution was normal. To adjust for the unknown distribution of the primary outcome and based on the lower bound for the asymptotic relative efficiency of the Wilcoxon test, the required sample size was increased by 20% to 36 patients. Further accounting for a dropout and loss-to-follow-up rate of up to 20%, consistent with the experience from previous studies of this type, a total sample size of 44 patients was required for inclusion in the trial.

The effect size of the expected difference which the study was powered for was aligned with the range of severity of sleep apnoea: the threshold between mild and moderate OSA is an AHI of 15 events/hour and the threshold between moderate and severe sleep apnoea is an AHI of 30 events/hour. We wanted to demonstrate that electrical stimulation was able to reduce OSA severity by at least 15 events/hour allowing for a drop in severity by one class (eg, the severe to the moderate range or from the moderate to the mild range).

### Statistical analysis

The differences in primary and secondary outcome measures between the ‘active treatment’ and ‘sham intervention’ were compared in a paired design (crossover trial) using SPSS Statistics (V.23; IBM, New York, USA). To compare study groups, the Wilcoxon (4%ODI) and paired t test for continuous paired variables were used. To identify predictors of response a stepwise multiple linear regression including the variables ‘age’, ‘gender’ and OSA severity (‘ODI’) was performed. The McNemar's χ^2^ test for nominal variables was used for the gender comparison in the responder group. The 95% CI was used to describe the treatment response. The level of significance was selected at p<0.05.

(For additional information on the methods and measurements, please refer to the online supplementary material).

## Results

### Patient characteristics

A total of 390 patients were assessed in order to determine the eligibility for this trial. Following initial assessment, 44 patients were further screened and underwent baseline polysomnography (first sleep study). Eight patients were excluded as screening failures and 36 patients were randomised in a crossover design and allocated to receive either active or sham treatment first. The participants returned for the first treatment night (second sleep study) after at least 3 days. After a ‘washout period’ of at least another 3 days, participants returned for the opposite treatment during the second treatment night (third sleep study). All 36 patients completed the trial after the third polysomnography and were included in the analysis (figure 1).

The studied patients were middle-aged, predominantly Caucasian male subjects and overweight-to-obese. The neck circumference was increased, the pharyngeal lumen was narrowed—only eight patients had a Mallampati score of I—and across the cohort there was a neutral waist-to-hip mass distribution. Participants were sleepy, as assessed by the ESS and had well-preserved lung function and normal daytime oxygenation. The past medical history indicated limited use of alcohol and nicotine (table 1). About 27.8% of patients had hypertension, 19.4% dyslipidaemia and 8.3% had type II diabetes.

**Table 1:** Demographic details of the patients included in the study (n=36)

Parameters	Data	Treatment first	Sham first	p Value
Age (years)	50.8 (11.2)	51.2 (11.9)	50.6 (11)	0.89
Sex (males/females)	30/6	13/1	17/5	0.22
White British, n (%)	29 (80.5)	12 (85.7)	17 (77.2)	<0.05
Caribbean, n (%)	3 (8.3)	1 (7.1)	2 (9.1)	0.83
African, n (%)	2 (5.5)	1 (7.1)	1 (4.5)	0.74
Indian, n (%)	1 (2.7)	0	1 (4.5)	0.41
White other, n (%)	1 (2.7)	0	1 (4.5)	0.41
Height (cm)	175.3 (8.4)	175.6 (6.4)	175.1 (9.6)	0.83
Weight (kg)	95.8 (17.7)	93.8 (17.7)	97.1 (18.1)	0.59
BMI (kg/m^2^)*	29.7 (26.9–34.9)	28.4 (26.6–35)	30.6 (27.4–34.9)	0.44
Neck circumference (cm)	42.6 (3.8)	42.0 (2.9)	42.9 (4.2)	0.42
Waist circumference (cm)	103.8 (16.5)	104.4 (14.1)	103.4 (18.2)	0.85
Hip circumference (cm)*	105.0 (99.0–111.0)	107.2 (99.8–111.0)	104.5 (98.3–108.0)	0.26
Waist:hip ratio	0.99 (0.08)	0.98 (0.07)	1.00 (0.08)	0.29
Mallampati score I, n (%)	8 (22.2)	4 (28.5)	4 (18.1)	0.56
Mallampati score II, n (%)	15 (41.6)	4 (28.5)	11 (50)	0.2
Mallampati score III, n (%)	8 (22.2)	5 (35.7)	3 (13.6)	0.12
Mallampati score IV, n (%)	5 (13.8)	1 (7.1)	4 (18.1)	0.35
ESS (points)	10.5 (4.6)	11.4 (4.6)	10 (4.5)	0.39
FEV_1_ (L)	3.13 (0.82)	2.94 (0.73)	3.25 (0.86)	0.26
FVC (L)	4.08 (0.89)	3.89 (0.86)	4.20 (1.07)	0.34
FEV_1_/FVC (%)	76.8 (8.2)	75.7 (9.9)	77.4 (7.0)	0.58
SpO_2_ awake (%)	94.6 (1.0)	94.7 (0.6)	94.5 (1.2)	0.43
Previous treatment: none/CPAP, n (%)	18 (50.0)/18 (50.0)	7 (50.0)/7 (50.0)	11 (50.0)/11 (50.0)	1.0
Alcohol consumption (units/week)*	2.0 (0.0–10.5)	1 (0.0–5.9)	4 (0–11.5)	0.49
Pack-years (years)* (12 lifelong non-smokers, 24 current or ex-smokers)	10.0 (5.0–14.3)	10.0 (6.5–12.1)	10.0 (5–15)	0.55

*Data were non-normally distributed and are expressed as median and IQR. Pack-years are calculated for current and ex-smokers only (n=24).

BMI, body mass index; ESS, Epworth Sleepiness Scale; SpO_2_: oxygen saturation.

### Baseline polysomnography

Participants had predominantly moderate-to-severe OSA with more severe upper airway obstruction in the supine posture, the majority of respiratory events being obstructive apnoea. Sleep was fragmented with an increased time of ‘wakefulness after sleep onset’ and reduced sleep efficiency. Normal sleep architecture was preserved, although rapid eye movement (REM) sleep latency was delayed. Snoring was observed during more than 10% of the night (table 2).

**Table 2:** Baseline polysomnography data of the studied patients (n=36)

Parameters	Results
ODI (events/hour)*	25.7 (16.0–49.1)
AHI (events/hour)*	28.1 (19.0–57.0)
Obstructive apnoea (events/hour)*	15.2 (6.7–31.4)
Central apnoea (events/hour)*	0.1 (0.0–0.5)
Mixed apnoea (events/hour)*	0.2 (0.0–1.9)
Obstructive hypopnoea (events/hour)*	7.8 (1.2–14.3)
Supine AHI (events/hour)	43.2 (27.0)
REM AHI (events/hour)	36.7 (24.9)
Arousal index (events/hour)	28.7 (14.8)
SpO_2_ asleep (%)	93.3 (1.6)
Nadir SpO_2_ asleep (%)*	80.5 (74.0–85.0)
Total sleep time (min)	337.5 (75.3)
Time in bed (min)	448.4 (51.8)
Sleep efficiency (%)	74.9 (15.1)
Sleep onset (min)*	18.8 (9.0–39.1)
Wake after sleep onset (min)*	79.0 (35.9–117.2)
REM latency (min)*	131.0 (69.5–162.5)
Sleep stage N1 (%)*	13.0 (8.3–18.7)
Sleep stage N2 (%)	50.7 (15.1)
Sleep stage N3 (%)	16.3 (12.8)
Sleep stage REM (%)	15.7 (9.3)
Snoring time (min)*	43.3 (15.2–72.4)
Snoring time (%)*	13.2 (5.5–22.3)

*Data were non-normally distributed and are expressed as median and IQR.

Sleep stages and snoring time are expressed as percentage of time asleep.

AHI, apnoea-hypopnoea index; N1–N3, non-REM sleep stages 1–3; ODI, 4% oxygen desaturation index; REM, rapid eye movement; SpO_2_, oxygen saturation.

### Primary and physiological outcome parameters

**Figure 2:**
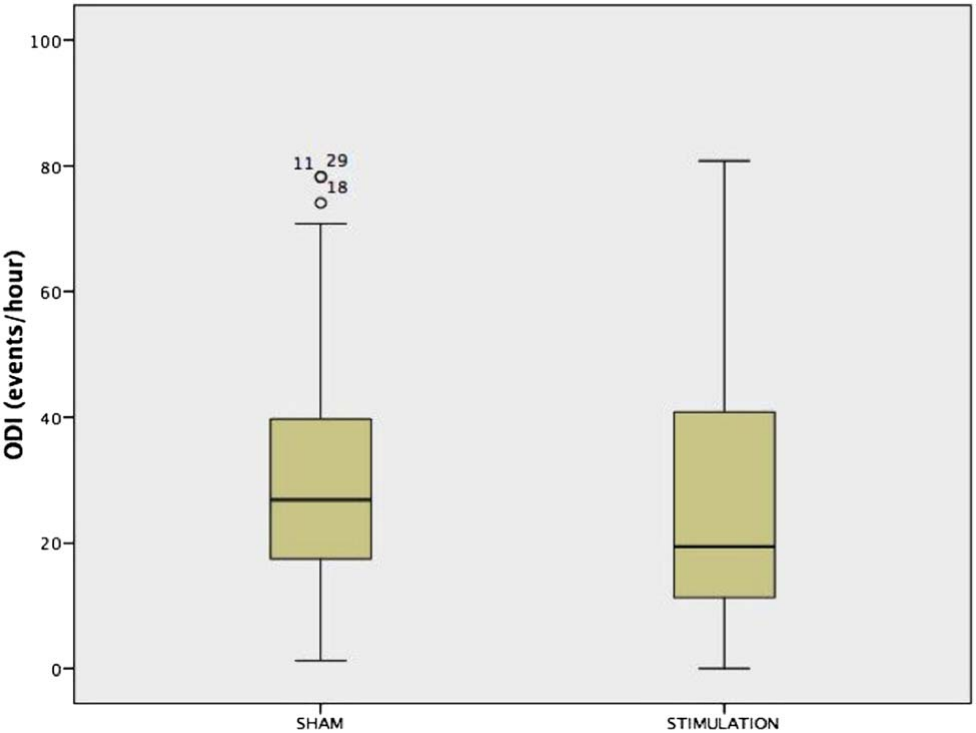
Box-and-whisker plot for the 4% oxygen desaturation index (4%ODI) in all studied patients (n=36).

**Figure 3:**
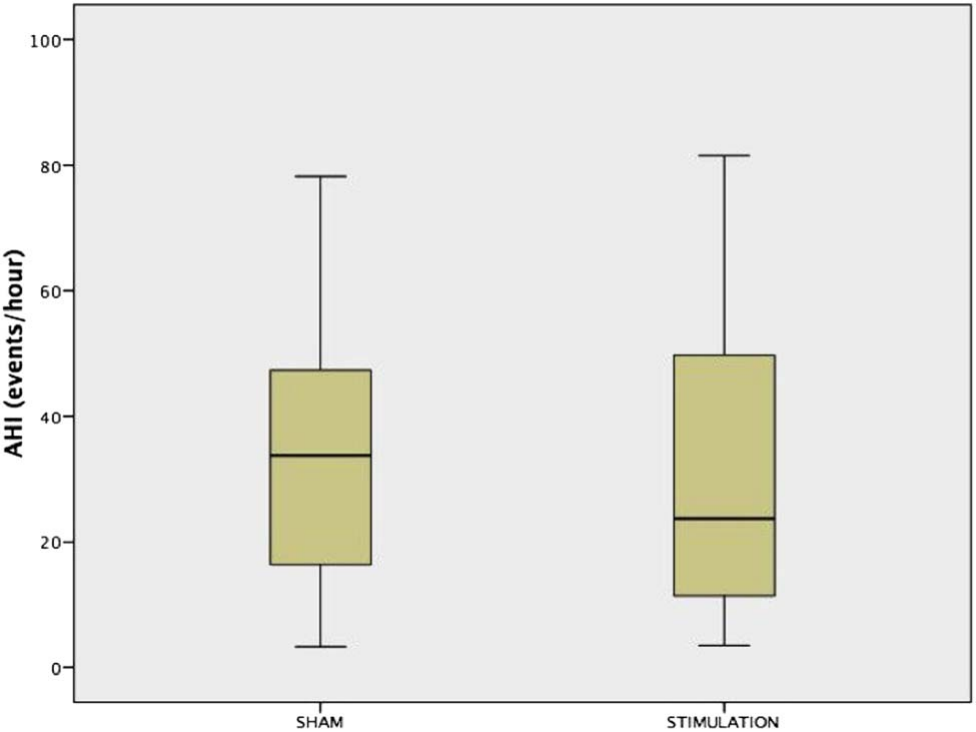
Box-and-whisker plot for the apnoea-hypopnoea index (AHI) in all studied patients (n=36).

During active treatment (current of 626.1 µA (409.8 µA)), the primary outcome of the trial, the ODI, improved modestly, with a mean of 4.1/hour (95% CI −0.6 to 8.9) for the whole group, when comparing with sham stimulation (figures 2 and 3). No differences were observed in the oxygenation levels and there were no significant improvements in the AHI or the supine AHI. Polysomnographic data revealed a similar sleep architecture and duration as observed at baseline with a reduction in N1 during the active treatment (table 3). The analysis of the apnoea:hypopnoea ratio revealed that the ratio was 1.59 (0.75–17.12) during the baseline sleep study and 0.82 (0.25–2.81) during the stimulation night (p<0.001), indicating a higher contribution of apnoea to the AHI without treatment.

**Table 3:** Respiratory and polysomnography data during sham treatment night compared with active treatment

Parameters	Sham stimulation	Active treatment	p Value
ODI (events/hour)*	26.9 (17.5–39.5)	19.5 (11.6–40.0)	0.026
AHI (events/hour)*	33.8 (16.6–46.1)	23.7 (11.4–47.6)	0.20
Obstructive apnoea (events/hour)*	9.9 (3.8–32.4)	7.6 (3.4–30.0)	0.21
Central apnoea (events/hour)*	0.5 (0.0–1.8)	0.4 (0.0–0.9)	0.53
Mixed apnoea (events/hour)*	0.5 (0.0–2.1)	0.1 (0.0–1.7)	0.68
Obstructive hypopnoea (events/hour)*	12.7 (3.0–23.6)	7.8 (4.5–15.6)	0.42
Supine AHI (events/hour)	44.9 (24.2)	38.6 (25.9)	0.09
REM AHI (events/hour)	35.2 (26.0)	31.3 (23.9)	0.50
Arousal index (events/hour)	28.8 (16.9)	22.6 (16.9)	0.007
SpO_2_ asleep (%)	93.2 (2.0)	93.2 (2.2)	0.48
Nadir SpO_2_ asleep (%)*	80.5 (74.5–86.0)	81.0 (74.0–84.0)	0.58
Total sleep time (min)*	366.3 (323.6–409.0)	356.8 (340.8–396.4)	0.41
Time in bed (min)*	452.0 (424.4–475.0)	447.3 (406.6–483.2)	0.77
Sleep efficiency (%)*	83.8 (70.9–89.3)	84.2 (69.1–89.0)	0.60
Sleep onset (min)*	14.5 (5.6–39.8)	17.0 (3.9–47.9)	0.17
Wake after sleep onset (min)*	51.5 (29.4–97.3)	52.3 (29.4–89.8)	0.52
REM latency (min)*	94.0 (64.1–156.6)	89.0 (64.0–132.0)	0.85
Sleep stage N1 (%)*	10.2 (7.0–20.3)	9.8 (6.3–15.0)	0.039
Sleep stage N2 (%)*	49.9 (40.0–58.8)	51.4 (43.1–65.6)	0.32
Sleep stage N3 (%)	15.7 (11.6)	15.4 (10.0)	0.76
Sleep stage REM (%)	16.9 (9.2)	16.0 (8.4)	0.50
Snoring time (min)*	64.6 (23.0–140.8)	46.3 (26.0–125.4)	0.45
Snoring time (%)*	19.9 (6.1–35.9)	17.0 (7.6–28.7)	0.47

*Data were non-normally distributed and are expressed as median and IQR.

AHI, apnoea-hypopnoea index; N1–N3, sleep stages; ODI, 4% oxygen desaturation index; REM, rapid eye movement sleep; SpO_2_, oxygen saturation.

### Secondary outcome parameters

The patients' device acceptance was good with patients reporting no skin discomfort or unpleasant sensations at night. There was no difference in patients' perceived sleep quality between the sham stimulation and the active treatment, but patients reported an improvement of their dry mouth after active treatment (table 4). The only significant side effect observed was one patient who complained about claustrophobia at night; this was during both treatment nights. The total count of mild side effects occurred in 2.8% of the studied cohort and there were no severe adverse events.

**Table 4:** Assessment of symptoms and side effects when waking after the sham stimulation and active treatment nights, as measured by a visual analogue scale (0–10 points)—higher scores indicate an improvement

Parameters	Sham stimulation	Active treatment	p Value
Feeling refreshed	5.7 (2.7–7.2)	6.6 (2.2–8.5)	0.40
Sleep quality	5.6 (2.9–7.1)	6.4 (2.4–8.0)	0.28
Mouth dryness	4.4 (2.2–8.5)	7.4 (4.9–9.7)	0.007
Tongue unpleasant sensation	9.9 (9.4–10.0)	9.9 (9.4–10.0)	0.63
Morning headache	9.4 (6.3–10.0)	9.9 (8.1–10.0)	0.27
Skin discomfort	9.9 (9.5–10.0)	9.9 (9.7–10.0)	0.95
Sleepiness*	3.0 (2.0–3.5)	3.0 (2.0–3.0)	0.29

*Sleepiness was assessed in the mornings using the Stanford Sleepiness Scale to pick up ad-hoc changes. All variables are presented as median and IQR.

### Responder group analysis

**Figure 4:**
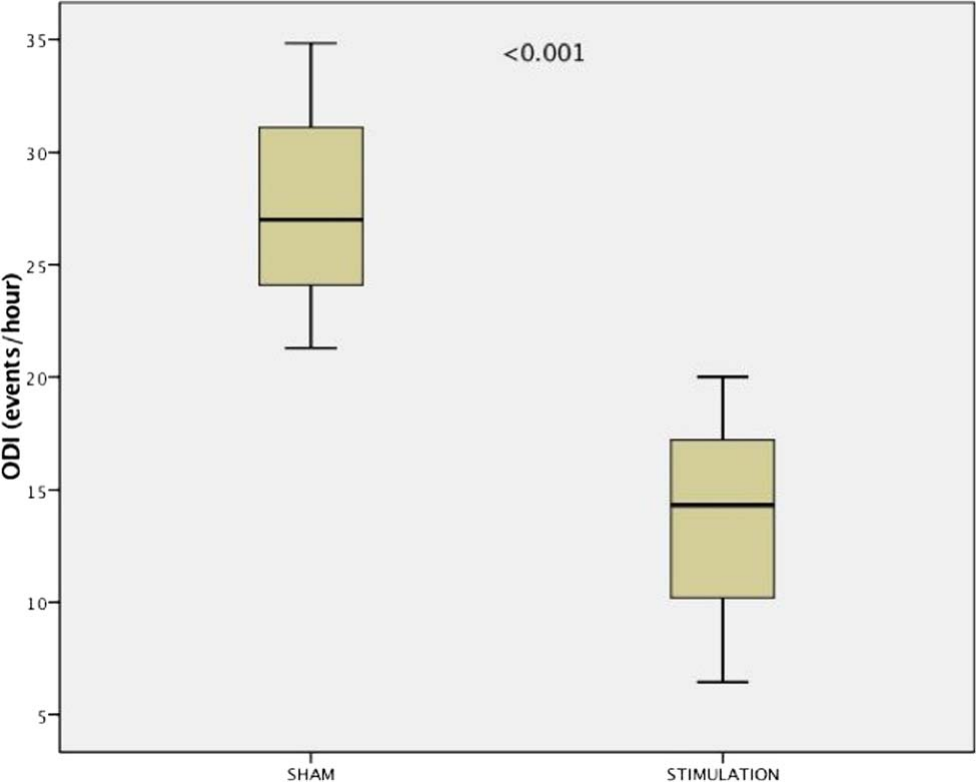
Box-and-whisker plot for the 4% oxygen desaturation index (4%ODI) among ‘responders’ (n=17). There is a significant improvement in the primary outcome between sham stimulation night and active treatment night.

**Figure 5:**
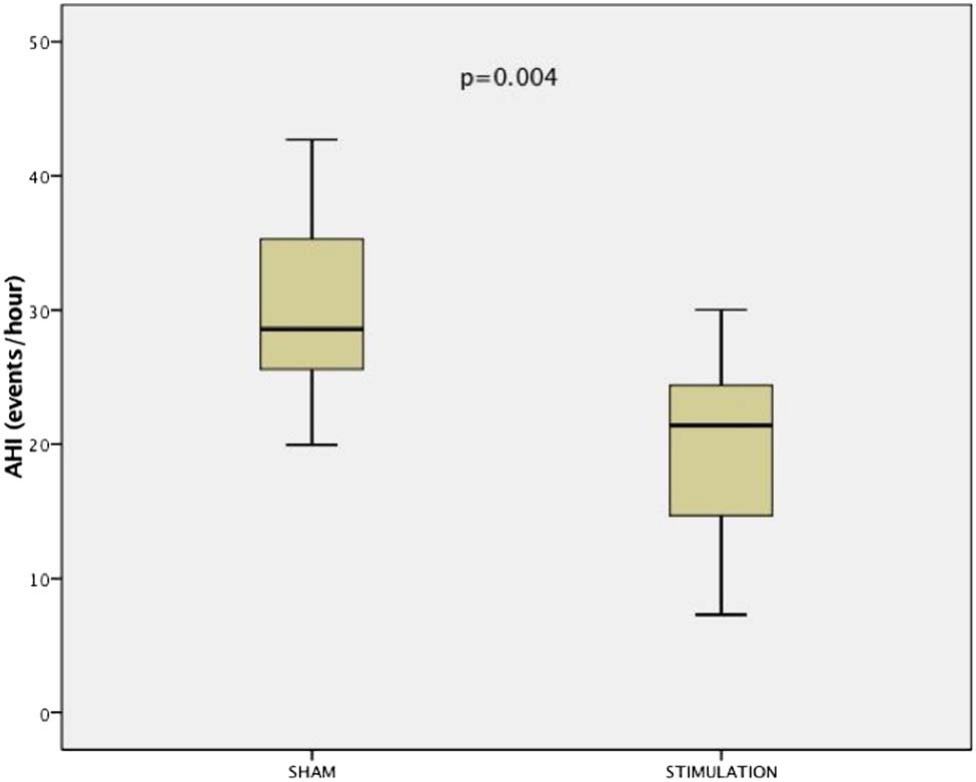
Box-and-whisker plot for the apnoea-hypopnoea index (AHI) among ‘responders’ (n=17). There is a significant improvement in the primary outcome between sham stimulation night and active treatment night.

While the primary outcome parameter improved modestly for the whole cohort, in 17 of 36 patients the ODI improved by >25% compared with sham night and/or the total ODI became <5/hour. This group had similar baseline characteristics as the whole cohort, but subjects were more likely to have mild-to-moderate OSA and to be of female gender (table 5). In these ‘responders’, 4%ODI improved by 10.0/hour (95% CI 3.9 to 16.0) (figure 4) and the AHI by 9.1/hour (2.0–16.2) (figure 5).

**Table 5:** Main characteristics of responders and non-responders

Parameters	Responders (n=17)	Non-responders (n=19)	p Value
Age (years)	48.4 (11.4)	53.0 (10.8)	0.22
Sex (males/females)	13/4	17/2	0.007
BMI (kg/m^2^)	32.3 (5.4)	30.0 (3.0)	0.19
Neck size (cm)	42.6 (2.8)	42.5 (4.5)	0.97
Mallampati score I, n (%)	3 (17.6)	5 (26.3)	0.53
Mallampati score II, n (%)	5 (29.4)	10 (52.6)	0.26
Mallampati score III, n (%)	6 (35.2)	2 (10.5)	0.07
Mallampati score IV, n (%)	3 (17.6)	4 (21.0)	0.63
Waist:hip ratio	0.98 (0.06)	0.99 (0.08)	0.67
ESS (points)*	10.0 (8.0–13.0)	13.0 (7.5–15.0)	0.14

*Data expressed as median and IQR.

BMI, body mass index; ESS, Epworth Sleepiness Scale; n, number.

In the total study cohort, there were 6 patients with mild OSA, all of whom responded; 13 patients with moderate OSA, with six responders (46.2%) and 17 patients with severe OSA, of whom five were responders (29.4%). There was a low negative correlation between ODI and the response to stimulation and the AHI and the response, respectively (r=−0.334, p=0.023 and r=−0.320, p=0.029, respectively). A stepwise multiple linear regression including the variables ‘age’, ‘gender’ and OSA severity (‘ODI’) at baseline identified only the ODI as an independent predictor for response (standardised β=0.348; 95% CI 0.012 to 0.372). The regression model with ODI severity at baseline predicted about 10% of the response variance (R^2^=0.121, adjusted R^2^=0.095, p=0.037). Age (p=0.851) and gender (p=0.720) were excluded from the model (see online supplementary material). There were no further significant correlations or predictors to identify responders.

A further subgroup analysis using more stringent criteria for treatment response revealed that 7 of the 36 patients (19.4%) had a reduction in the AHI of >50% when compared with sham stimulation. Out of this group, 2 of the 36 patients (5.6%) had a residual AHI <10/h during electrical stimulation and a total of 6 of the 36 patients (16.7%) had an AHI <15/hour, which would indicate mild OSA. In the responder group, 2 of the 17 patients (11.7%) had an ODI <5 events/hour during sham treatment. When taking the data of these two patients out the Wilcoxon signed-rank test, there was still a significant difference between the two groups (Z-value −3.4078, p=0.00064).

## Discussion

This is the first randomised, sham-controlled and double-blind clinical trial testing the feasibility of transcutaneous electrical stimulation of the pharyngeal dilator muscles in OSA for a whole night. The primary outcome, improvement in the ODI, revealed a modest improvement for the whole group compared with sham stimulation. The majority of the participants improved their sleep-disordered breathing and almost half of the studied participants were identified as responders with an improvement by a clinically relevant margin. Although the total AHI did not change during stimulation, there was a shift in the ratio of obstructive apnoea:hypopnoea when electrical current was applied. This observation indicates a resolution of the upper airway obstruction during apnoeas with electrical stimulation.

Secondary outcome parameters were related to patients' perception of stimulation and sleepiness, snoring, oxygen saturations and side effects. Patients' sleep quality, polysomnographically determined sleep architecture and sleep duration were not adversely affected by the use of electrical stimulation. There were no significant adverse events during the trial and patients did not experience any pain or skin discomfort. Claustrophobia was observed during two nights in a single patient (sham and true stimulation night). Snoring, average and nadir oxygen saturations were not altered by stimulation in this cohort; however, participants experienced fewer problems with a dry mouth following the night with electrical stimulation.

### Clinical relevance of transcutaneous stimulation

Electrical stimulation has long been studied as a technique to influence upper airway dilator function in sleep. Prior studies have reported both positive^11^ ^12^ ^17^ and negative^13^ ^14^ results, likely to reflect the heterogeneous populations studied and different approaches used.^7^ An important point when using this technique is to avoid arousals from sleep. For many years, no further reports were published using the transcutaneous technique. Following on from our own feasibility study,^15^ the current trial used a titration algorithm during wakefulness to define individual skin sensation thresholds of electrical current to enable unproblematic nocturnal use of currents within the range observed to be comfortable while awake.

CTES rather than intermittent or inspiratory-triggered stimulation bursts is less likely to trigger uncomfortable skin sensation, as changes in current intensity activate specific skin receptors; CTES has the advantage of working with a single-channel device (‘pacing’) without the need for ‘sensing’. The use of continuous low current requires less force to maintain the neuromuscular tone in the upper airway than intermittent stimulation as it is easier to maintain upper airway patency than to initiate the reopening of an occluded airway. It is important that this approach can be used for prolonged periods in patients with sleep apnoea with no apparent adverse effect on sleep quality or sleep duration.

### Hypoglossal neural stimulation

Several recent studies have investigated the use of an implantable electrical stimulator to target the distal branch of the hypoglossal nerve that innervates the genioglossus.^8–10^ ^18^ ^19^ The Clinical Trial by Apnex Medical (Roseville, Minnesota, USA; NCT01446601) was terminated prematurely because the primary outcome, a reduction in OSA severity (defined as AHI reduction >50% and AHI <20 and ODI 4% reduction ≥25% or ODI 4% <5 from baseline to 6-month follow-up) was not met. In contrast, the STAR trial achieved positive results.^10^ An important feature of the STAR trial was sophisticated screening to identify potential ‘responders’ to the treatment. The investigators excluded patients with pronounced anatomical abnormalities of the upper airway and those with concentric collapse of the retropalatal airway, as assessed by endoscopy during drug-induced sleep.^10^ After screening 929 patients, 126 participants had a device implanted and 124 completed the trial. At 12 months the median AHI was reduced by 68% from 29.3/hour to 9.0/hour. In a randomised therapy-withdrawal arm the features of sleep apnoea were again observed when the treatment was discontinued (AHI 25.8/hour; ODI 23.0/hour). In the STAR trial, 56 patients were excluded from implantation following endoscopy during drug-induced sleep and 13 patients were excluded because of anatomical abnormalities of the upper airway following surgical consultation.

Compared with hypoglossal nerve stimulation, the effect size of CTES in the current trial is smaller. Several factors can explain this observation: the delivery of effective electrical current to the muscles is affected by skin and soft tissue resistance; in patients with large neck circumference, this can be a significant limitation. Stimulation frequency, duration and waveform need to be considered when stimulating for longer periods to maintain force generation while avoiding muscle fatigue. Last, the intensity of the electrical current is dependent on individual comfort and perception and titration of the current needs to be tailored to patient's individual perception of skin discomfort to avoid arousal from sleep. Future studies using transcutaneous electrical stimulation will need to consider the level of obstruction of the upper airway to identify potential responders prior to enrolment in trials. Transcutaneous electrical stimulation might also be used to test patients prior to implanting hypoglossal nerve stimulators to test the individual response.

### Responders to transcutaneous electrical stimulation

In the current trial, 17 of the 36 participants (47.2%) had improvement in OSA severity during the electrical stimulation night, as defined by the ODI and the AHI.^10^ From the studied cohort, it appears that this method is more suitable for milder disease and in female subjects. In the multivariate analysis none of the studied characteristics or demographics seemed to be associated with responsiveness. The neck anatomy and the distance between dilator muscles and dermal patches as well as the threshold of comfort for the effective current are likely to be further determinants of effectiveness.

### Effect on REM sleep AHI

In REM sleep, the peripheral skeletal muscles are in a state of physiological atonia during which the work of breathing is predominantly delivered by the diaphragm. The tone of the upper airway dilator muscles is reduced and the critical occlusion pressure can significantly increase making it more likely that the subjects experience obstruction and collapse of the upper airway as the positive intramural and the negative inspiratory pressure gradients favour unopposed occlusion.

It is therefore of interest to understand how transcutaneous electrical stimulation impacts on the stability of the upper airway, as indicated by the AHI. For the whole group, the REM-sleep related AHI did not significantly improve (REM AHI for sham stimulation 35.3 (26.0) versus active treatment 31.3 (23.9)/hour, p=0.50) and this might indicate that the force to maintain upper airway patency is not strong enough in REM sleep due to the increased load and reduced endogenous neuromuscular tone. However, neither ‘responders’ nor ‘non-responders’ improved their REM AHI significantly with stimulation (p=0.282 for responders, p=0.725 for non-responders), but responders had a lower REM AHI than non-responders with active stimulation (non-responders REM AHI 36.9 (13.3–56.8) versus responders REM AHI 13.3 (6.7–32.7)/hour, p=0.044). This is consistent with the observation that patients with milder forms of sleep apnoea who were less obese were more likely to be responders.

### Subjective perception of transcutaneous electrical stimulation

Previous studies using transcutaneous electrical stimulation for the treatment of sleep apnoea did not apply electrical current for the whole night. The TESLA data on patients' perception importantly highlight the feasibility of the method, with patients remaining asleep with no difference in their sleep profile compared with sham stimulation. Sleep quality was not adversely affected. Following the sham stimulation, patients complained more about a dry mouth in the morning than after active treatment. Whether this is a feature of a more patent upper airway at night or the mouth being closed remains to be elucidated.

### Snoring in patients with sleep apnoea

The current trial failed to reduce snoring duration during stimulation nights in patients with sleep apnoea. The breakdown of the AHI shifted during electrical stimulation, the obstructive apnoea:hypopnoea ratio during the baseline sleep study was 1.59 vs 0.82 during active treatment. This indicates a preferential resolution of apnoea with electrical stimulation. However, continued flow-limited breathing during ongoing hypopnoea is likely to contribute to the total time of snoring. In comparison, there was absence of airflow during obstructive apnoea and no snoring sound. Whether snoring in patients with milder sleep apnoea, patients with upper airway resistance syndrome or snorers without flow limitation would improve using this treatment remains to be studied.

### Stimulation pattern

The way electrical current can be used to stimulate the upper airway dilator muscles transcutaneously is influenced by multiple factors. Waveform (rectangle, triangular, single impulse, rounded), polarity (unipolar, bipolar), frequency, intensity and duration (continuous, intermittent, triggered) are important factors to generate efficient force and avoid muscle fatigue. Following on from previous work, the current trial used bipolar current, individually titrated by daytime in awake patients to define the lower and upper current thresholds of skin sensation at a frequency of 30 Hz.^15^ Inspiratory-triggered stimulation has the advantage of avoiding fatigue but requires additional recording for sensing of a physiological signal to identify inspiration (eg, flow, sound, inspiratory effort measuring either movement or electromyography activity), live analysis and sophisticated algorithms to stimulate the muscle at the right time. In contrast, continuous stimulation, as previously used, is effective in avoiding these problems; similar to CPAP therapy, the treatment is provided all night but it is likely to cause muscle fatigue. The stimulation pattern used for the TESLA trial (5 s on/5 s off) was chosen following initial studies to address these points; it does not require additional sensing and its duty cycle will guarantee stimulation during any potentially occurring apnoea which is defined as absence of airflow for >10 s. This pattern also provides sufficient rest time for the muscle to avoid fatigue.

### Limitations

Upper airway endoscopy during drug-induced sedation is presently not standard practice in the UK, but this technique might be of theoretical value in selecting patients for this therapy by describing the level of upper airway obstruction. Endoscopy was not considered when screening patients for the current trial and a better characterisation of the upper airway would likely have led to a greater number of ‘non-responders’ being prospectively excluded. Future trials using transcutaneous electrical stimulation could screen for likely ‘responders’, define patient phenotypes, test the feasibility and effectiveness of this method in the community and test whether it is sufficient to treat REM-related events. Endoscopy could also describe the site of upper airway collapse during delivery of transcutaneous electrical current. It is important to identify the impact of posture in this method and whether a treatment effect is observed in the non-supine posture. Determinants of effectiveness like posture and neck flexion should be studied in future. These points are important in the context that 2 of the 17 responders had a normal ODI in the sham night. Whether this improvement compared with the baseline sleep study is due to the taping of the submental region is unclear, but changes in the neuromuscular tone due to increased afferent feedback could, in part, contribute to our findings. This study was set up to test transcutaneous electrical stimulation for a single night only and it remains to be shown whether use of dermal patches and transcutaneous stimulation over longer follow-up periods is a feasible method and whether responders benefit symptomatically.

The double-blind study design of the TESLA trial was chosen to minimise bias caused by patients' or research staff perception of the method. A computer mode provided random selection of active treatment or sham stimulation. Once the mode was selected, the computer indicated at night that stimulation was delivered, independent of whether it was sham stimulation or active treatment, to simulate a potential stimulation artefact. Independent experienced technicians from the sleep laboratory were assigned for the offline analysis after the patients had been studied over all three nights. The data were then inserted in the database by a different investigator, still in the randomised order and without access to the respective raw data of the polysomnography. After the last patient had been studied and data acquisition had concluded, the data were unblinded in that the sham stimulation nights' data were separated from the active treatment nights' for analysis by the medical statistician. Any potentially small stimulation artefact picked up in the technicians' analysis of the original polysomnographies would not have been reported to the trial's team or the patient and is therefore unlikely to have impacted on the trial's outcome.

Only about 1 in 10 screened patients underwent randomisation and, therefore, the sample of the randomised patients does not fully represent the whole cohort of patients with OSA. However, it is not uncommon that strict adherence to a protocol requires screening of a large number of patients who, eventually, turn out to be excluded. In the STAR trial, Strollo *et al*^10^ conducted a randomised controlled trial of invasive electrical stimulation in patients with OSA and they recruited 929 subjects, of whom only 126 were randomised and included in the treatment group (13.6%). Broader inclusion criteria could help the generalisability of observed effects. However, particularly in studies testing a novel therapeutic approach, the inclusion of inappropriate patients could dilute the effect size, as it is likely that these patients are less responsive. It should be pointed out that this approach will not replace CPAP as the standard first choice therapy for OSA, but might benefit some patients who are unable to wear CPAP masks. Importantly, even patients who are well established on standard treatment and compliant with CPAP may wish to try novel and non-invasive alternatives.^20^ The prevalence of OSA is rising and even a modest response using electrical stimulation could be helpful in avoiding symptoms and long-term risks in some of the patients who fail CPAP therapy. However, it is important to note that the present study was not powered to identify predictors of response and, therefore, the post-hoc results of the subgroup analysis must be interpreted with caution.

## Conclusion

Transcutaneous electrical stimulation of the upper airway dilator muscles in OSA can be safely delivered throughout the whole night. Although we observed only a modest improvement in the whole study cohort, approximately half of the studied population were ‘responders’—predominantly those with mild-to-moderate disease. Future use of this method should focus on the prospective identification of responders. Defining upper airway features of those who could benefit and assessing long-term symptomatic improvements and feasibility of the method in the domiciliary setting will be important to further develop this approach.
