# Supplementary material for: Randomised sham-controlled trial of transcutaneous electrical stimulation in obstructive sleep apnoea
Source: Thorax. 2016 Jul 19;71(10):923–31. doi: 10.1136/thoraxjnl-2016-208691 (PMC5036236; doi:10.1136/thoraxjnl-2016-208691)
Supplement: Supplementary material [file thoraxjnl-2016-208691supp.pdf]

Online Data Supplement

Randomised sham-controlled trial of transcutaneous electrical stimulation in obstructive sleep apnoea

Martino F Pengo, Sichang Xiao, Culadeeban Ratneswaran, Kate Reed, Nimish Shah, Tao Chen, Abdel Douiri, Nicholas Hart, Yuanming Luo, Gerrard F Rafferty, Gian Paolo Rossi, Adrian Williams, Michael I Polkey, John Moxham, Joerg Steier

Patients and Methods

We enrolled patients referred to Guy`s and St Thomas` NHS Foundation Trust and the Royal Brompton and Harefield NHS Foundation Trust sleep services (both London, UK) for treatment of OSA between 03/2013 and 12/2015. The patients underwent home-based nocturnal pulse oximetry before being seen in the service. The research team approached those patients with a nocturnal pulse oximetry test and a symptom score (Epworth Sleepiness Scale, ESS) suggestive of OSA and informed them of the study (pre-screening). All patients were provided with a patient information sheet and informed written consent was obtained prior to enrolment.(E1)

Inclusion and exclusion criteria

The study included patients aged 18–75 years with a body-mass index (BMI) between 18–40 kg/m^2 who had OSA with an oxygen desaturation index (4%ODI) ≥15/hour or with an ODI ≥5/hour plus an Epworth Sleepiness Scale score >10 points. Exclusion criteria were obesity–hypoventilation syndrome (total sleep time with SpO_2<90% of more than 10% of the night or pCO_2>6kPa), smoking history of >20 pack years (or significant airway obstruction with an FEV_1/FVC ratio <0.7), acute or critical illness, acute psychosis or chronic mental disorder affecting capacity, previous domiciliary non-invasive ventilation or metal implants in the upper part of the body (including pacemakers, excluding dental implants). Patients on CPAP therapy were asked to discontinue the treatment for more than one week prior to the baseline sleep study. Patients were withdrawn from the trial if severe adverse events were reported. Their data were also excluded if they withdrew consent or lost capacity to give informed consent.

Recruitment, selection and study procedures

Patients who were referred for investigation of obstructive sleep apnoea were approached by the study co-ordinator or the clinical research fellow of the TESLA trial. During this first contact, the patient was given an information sheet and the study co-ordinator/clinical research fellow indicated

that they would contact any potential future participant >24h later to provide sufficient time to consider their decision and provide an opportunity to answer any questions about the trial. If the patient agreed to participate another appointment was made. Informed and written consent was obtained at this point following sufficient time for questions.

The eligibility criteria were assessed prior to enrolment. Inclusion criteria were male and female subjects, age >18years and <75years, body-mass index (BMI) >18 and <40kg/m^2, current non-smokers, sleep apnoea with an oxygen-desaturation-index (4%ODI) ≥15/hour or sleep apnoea with an ODI ≥5/hour plus an Epworth sleepiness score >10 points. Exclusion criteria were morbid obesity (BMI>40kg/m^2) or cachexia (BMI<18kg/m^2), obesity-hypoventilation syndrome (total sleep time with SpO_2<90% of more than 10% of the night), active smokers with a smoking history of >20pack years, acute or critical illness, acute psychosis or chronic mental disorder affecting capacity, previous home-mechanical non-invasive ventilation and metal implants in the upper part of the body (this excluded dental implants). Patients on previous CPAP therapy who wished to participate were enrolled following discussion with the clinical team.

Enrolled participants were admitted for a diagnostic baseline full polysomnographic study. Demographics and baseline measurements were recorded: past medical history, comorbidities, medication, smoking history, spirometry, arterial blood gas analysis, height, weight, BMI, waist/hip and neck circumference, Mallampati score and abdominal skin double-layer thickness. During the admission for the diagnostic sleep study, the patients were introduced to the stimulation technology to familiarise and to determine the lowest felt and the highest tolerated electrical current to define the range of stimulation current that could be used later during the interventional studies (Figure E1A+B).

Following the baseline diagnostic full polysomnographic study (AHI≥15/hour or AHI≥5/hour plus Epworth score>10) the patients were randomised into two arms: In arm (A) the patient underwent active treatment during the first study night followed by the second night using sham-treatment. In arm (B) the patient would undergo a sleep study with sham-stimulation during the first night followed by a second night with active treatment. There was a “washout period” of at least three days between the two interventional sleep studies.

Following the titration of the current whilst awake, the patients would spend the first night of treatment arm (A) or arm (B) in the sleep laboratory and a full polysomnographic study was carried out (commencing no later than 22.00h). In the morning (07.00h) the patient were woken up and

assessed for sleepiness and subjective discomfort (Stanford sleepiness and visual analogue scales). The patients were then discharged home for the "washout period" of at least 3 days to guarantee no lasting effects caused by the first study night. They were invited back for the second interventional study night according to the crossover design for arm (A) or arm (B), commencing no later than 22.00h. In the morning assessment of sleepiness and comfort were repeated (07.00h). Once the end of the study was reached patients were referred back to the clinical sleep service for further care and follow up.”

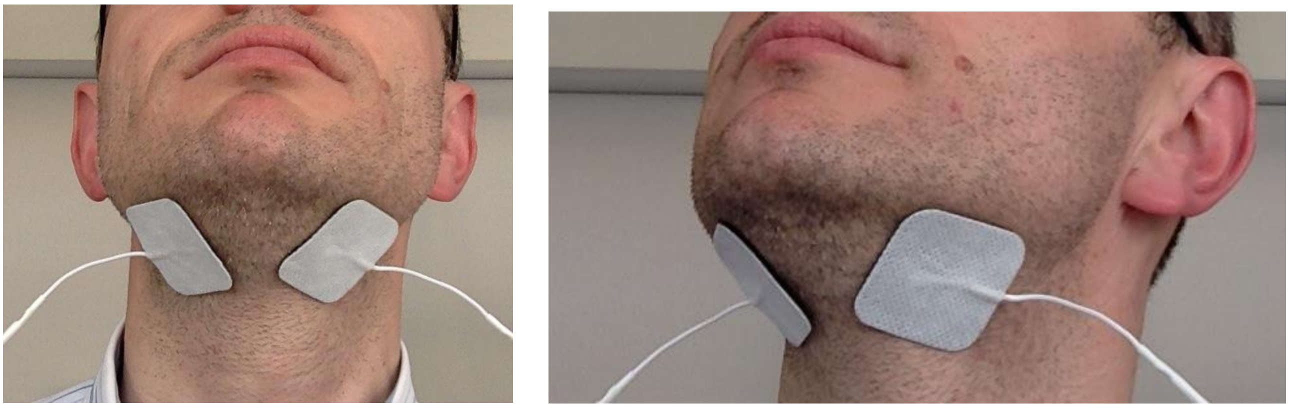

Figure E1A+B: Frontal and lateral view of the placement of the transcutaneous electrical stimulation patches (4 x 4cm), as described earlier (Steier *et al*, Chest 2011).

Baseline assessment

Following inclusion in the trial and informed written consent, the patients included in the study underwent a baseline assessment. Demographics and baseline measurements were recorded, including past medical history, comorbidities, medication, smoking history, height, weight, body mass index (BMI), waist/hip and neck circumference and Mallampati score. Spirometry (Carefusion Micro I, Micromedical, Basingstoke, UK) was performed in accordance with current guidelines.(E2, E3) Sleepiness was assessed at baseline using the ESS.(E4) The following questions were asked following each of the interventional nights after waking the patient in the morning:

- Do you feel refreshed ?
- How was the quality of your sleep ?
- Is your mouth dry ?
- Do you feel any pain/unpleasant sensation in your tongue ?

- Do you have any morning headache ?
- Do you have any skin discomfort close to the stimulation patches ?

Each answer was marked on a visual analogue scale (0-10cm) with higher values indicating favourable outcomes.

Polysomnography

Patients underwent nocturnal full polysomnography (Alice 5® equipment, Respironics®, Murrysville, PA/USA, or Embla® RemLogic, Embla Systems, Ontario, Canada) at baseline and during randomly allocated sham- and active electrical stimulation nights. The randomization sequence was generated using GraphPad Software QuickCalcs (www.graphpad.com/quickcalcs/randomize1.cfm; GraphPad Software Inc, La Jolla, CA/USA) and concealed from the study team until patients were consented and assigned the respective sequence of nights. The three study nights were recorded with a gap of at least 3-days to provide 'wash-out' periods.

The nocturnal recordings included the electro-encephalogram (EEG) according to the ten-twenty system (C3-M2, C4-M1, O2-M1) and the AASM recommendations.(E5) Electrooculography (EOG) was performed to detect eye movements (E1-M2; E2-M2). An electromyogram (EMGchin) was recorded from the submental region after the skin had been prepared with alcohol wipes and Nuprep skin prep gel (Nuprep, DO Weaver & Co, Aurora/CO, USA). Body position was recorded via a body position sensor (1.5mm, Alice 5, Respironics®, Murrysville, PA/USA) that was attached to the body to record body posture (see online supplement for method); it was further by the technician attending the overnight recording. Pulse oximetry was used to sense oxygen saturation and pulse frequency. Airflow was measured via an oronasal thermistor, and nasal pressure was recorded with a nasal tube. Abdominal and chest wall movements were detected via uncalibrated inductance plethysmography bands around the chest and abdomen. Snoring was recorded with a microphone. Limb movements were detected with surface electrodes. An apnoea event was defined as the drop in the airflow by ≥ 90% of pre-event baseline using an oronasal thermal sensor for ≥ 10 seconds. Hypopnoea was scored when the peak signal excursions dropped by ≥ 30% of pre-event baseline for ≥ 10 seconds in association with either ≥ 3% oxygen desaturation or an arousal. Hypopnoea was also scored in case of a ≥ 4% oxygen desaturation from pre-event baseline. Obstructive events were scored when respiratory movement was seen, represented by the excursions of the thoracic and abdominal bands. A central event was scored when no respiratory movement was seen. Respiratory effort-related arousal (RERA) was defined as a respiratory effort

associated with an EEG arousal without evidence of oxygen desaturation or significant airflow reduction. All respiratory events were scored according to the current American Academy of Sleep Medicine (AASM) criteria.(E6)

Full polysomnography began no later than 22.00 h and ended around 07.00 h. The patients were woken and assessed for sleepiness (Stanford Sleepiness Scale ranging from 1-7) (E7) and subjective discomfort (a seven-points, self-rated visual analogue scale ranging from 0-10). Different domains were assessed, including neck and tongue discomfort, dryness of the mouth, sleep quality, feeling refreshed and morning headache. Blood pressure was recorded before and after each sleep study, with an automated device (Omron MX3 Plus, Kyoto, Japan). After the final sleep study and assessment the patients were transferred to the regular sleep service for further clinical care and follow-up.

Transcutaneous electrical stimulation

The stimulator was controlled by a computer using a specially developed software package (Sleep Apnoea GUI, MIAT®, Petersfield/UK). Besides a live mode to titrate currents when applying the patches in the evening and testing subjective sensation thresholds of discomfort (these were marked as the maximal limits of current used at night) the software allowed for blind selection of either sham- or active stimulation mode to guarantee a double-blind study design.

Following randomisation and prior to the two interventional sleep studies, participants were introduced to the technology for familiarisation and to determine the highest tolerated currents to be used for the overnight studies. During the two interventional polysomnographies (active treatment and sham stimulation), the automated stimulation software was activated when the patient first reached stage N2 sleep and electrical current or sham stimulation was delivered in a 5s-on and 5s-off mode until the device was turned off in the morning.

Titration of stimulation current

Before using electrical current in human beings whilst asleep it is important to consider potential sensation (pain, tingling), frequencies (force/fatigue, arrhythmic potential), current (intensity of tissue penetration) and pulse shapes (sensation/force). Following on from our experience in the feasibility study (Steier *et al*, Chest 2011) (1) we commenced titrating the stimulation current in awake participants following explanations and consent. We started a titration ramp from 0mA and slowly increased the current to the first sensation threshold (mPL1) when the patient first noticed any sensation. We marked this level of current and then slowly increased the current until the

patient felt that it became uncomfortable (MTL1). This threshold was again noted and the current was then decreased until the patient could not feel it any longer (mPL2). Eventually, the current was slowly increased again until the patient felt it became uncomfortable (MTL2). An average of the lower sensation threshold ((mPL1+mPL2)/2) and an average of the maximally tolerated level of current ((MTL1+MTL2)/2) were calculated and the maximal current for usage in the asleep patient was set to half the current between these averages (Figure E2). This titration was undertaken prior to any interventional sleep study.

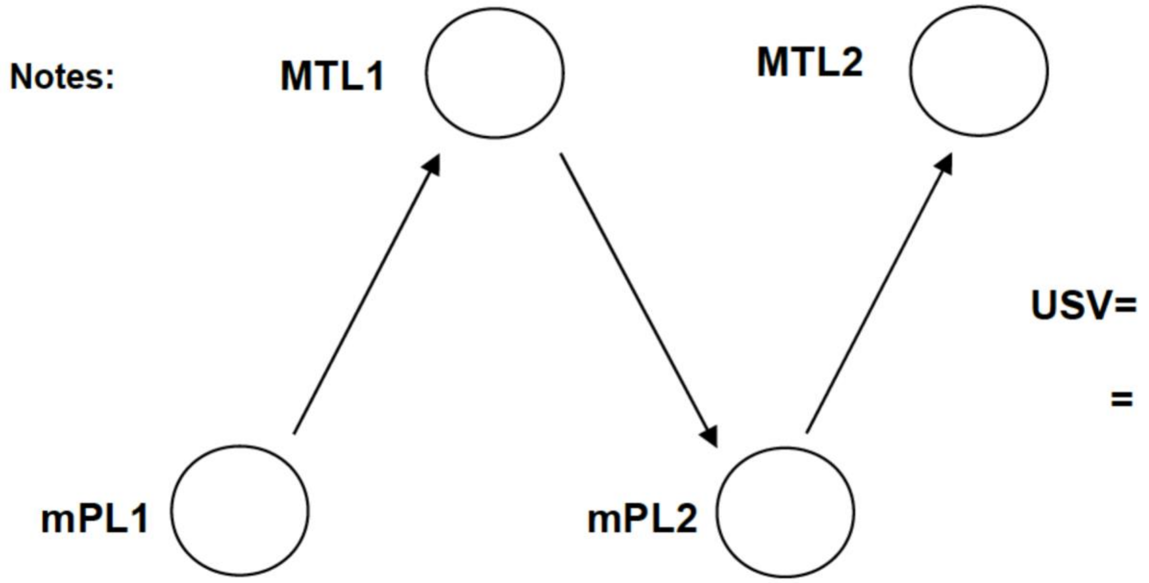

Figure E2: Copied from the case report form (CRF) of the TESLA trial to define the maximal current used in the asleep patient. MTL=maximum tolerable level, mPL=minimum perceptible level, USV=used stimulation voltage. The indices 1 and 2 were used to differentiate between repeated measurements.

Outcome Measures

The primary outcome measure for this trial was the 4%ODI per hour of sleep (events/hour). The 4%ODI was chosen as the primary outcome parameter in preference to the apnoea-hypopnoea index (AHI) because electrical stimulation can lead to an incomplete re-opening of the upper airway during an ongoing apnoeic event which, hypothetically, may result in a nominal increase in the AHI. Incomplete upper airway re-opening could, hypothetically, convert one long apnoeic event (complete cessation of airflow) into several shorter hypopnoeas (with limited airflow). As a consequence, the total number of apnoeas and hypopnoeas (i.e. the AHI) would increase. Due to a

longer circulatory delay the 4%ODI is less likely to be affected by incomplete reopening of the upper airway.

Sample size calculation

A sample size calculation for the primary outcome, 4%ODI, was performed based on the results of a previous feasibility study.(E8) A sample size of 30 patients would achieve 90% power to detect a mean of paired differences between the two treatments of 17.9 events/hour with an estimated standard deviation of differences of 18.9 events/hour and with a significance level of 5% using a two-sided Wilcoxon test assuming that the actual distribution was normal. To adjust for the unknown distribution of the primary outcome and based on the lower bound for the asymptotic relative efficiency (ARE) of the Wilcoxon test, we increased the required sample size by 20% to 36 patients. Further accounting for a dropout and loss-to-follow-up rate of up to 20%, consistent with the experience from previous studies of this type, a total sample size of 44 patients was required for inclusion in the trial.

Statistical analysis

The differences in primary and secondary outcome measures between the “active treatment” and “sham intervention” were compared in a paired design (cross-over trial) using SPSS® Statistics Version 23 (IBM®, NY/USA). Variables were tested with Shapiro-Wilk test and were expressed as median and interquartile range (IQR, 1st to 3rd quartile) if non normally distributed or as mean and standard deviation (SD) if normally distributed. For categorical variables (sex, Mallampati score, smoking habit, ethnicity, previous CPAP use) frequency counts and percentages are presented. To compare study groups, the Wilcoxon (4%ODI) and paired t-test for continuous paired variables, and the chi-square test for categorical variables were used. To identify predictors of response a stepwise multiple linear regression including the variables “age”, “gender” and OSA severity (“ODI”) was performed. The level of significance was selected at $p<0.05$.

Results

In the diagnostic polysomnography of the studied cohort there was a non-significant effect of posture on the AHI (AHI 28.1 (19.0-57.0) *vs* supine AHI 43.2 (27.0) /hour, p=0.303) and there was no significant difference in the time spent supine (p=0.205). The supine AHI did not significantly differ between sham-night and active treatment (Figure E3).

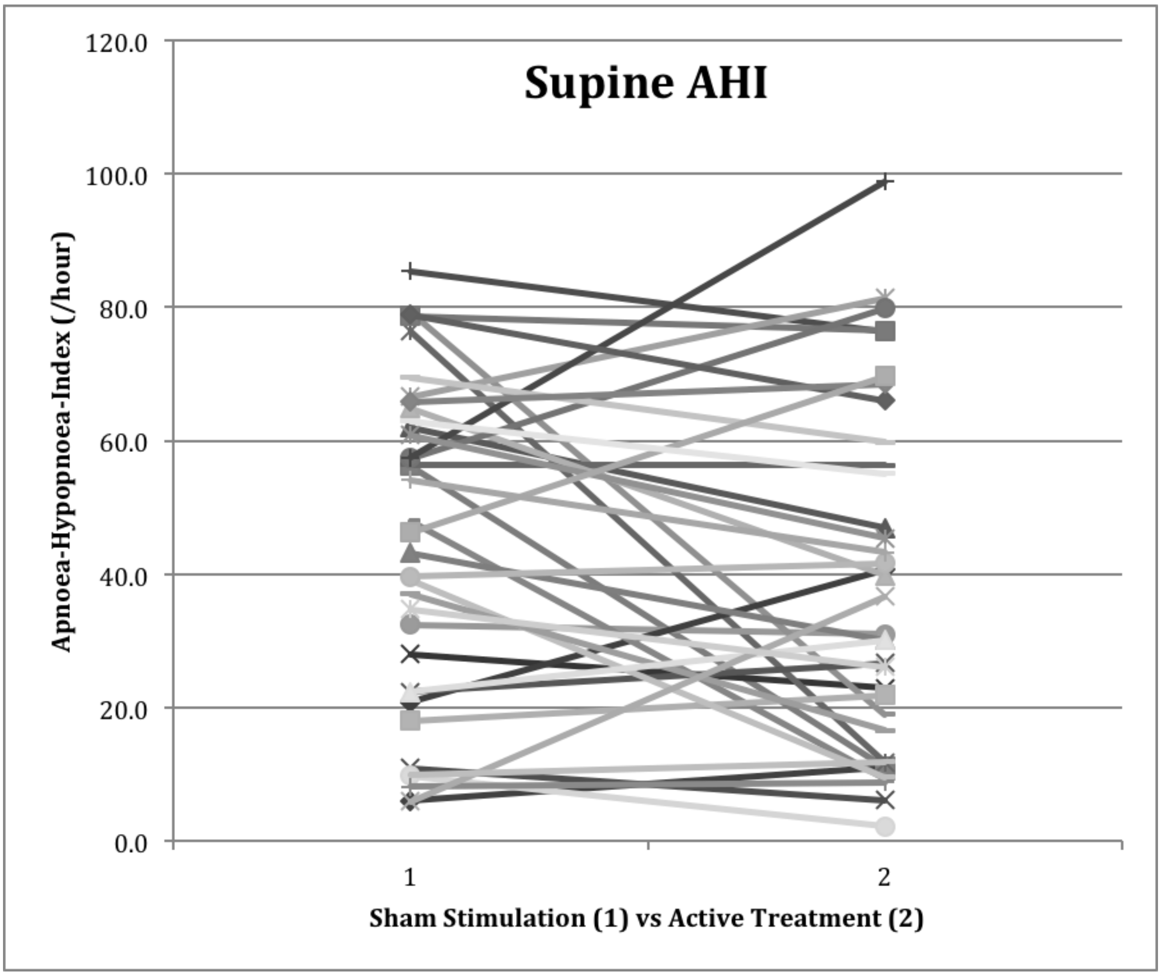

Figure E3: Supine apnoea-hypopnoea-index (AHI) for sham stimulation (1) *vs* active treatment (2). There was no significant improvement between the two nights (supine AHI during sham stimulation 44.9 (24.2) *vs* active treatment 38.6 (25.9) /hour, p=0.09. Results expressed as mean (SD).

Individual changes in the 4%ODI (figure E4) and the AHI (figure E5) plotted for the responder group indicate significant improvements.

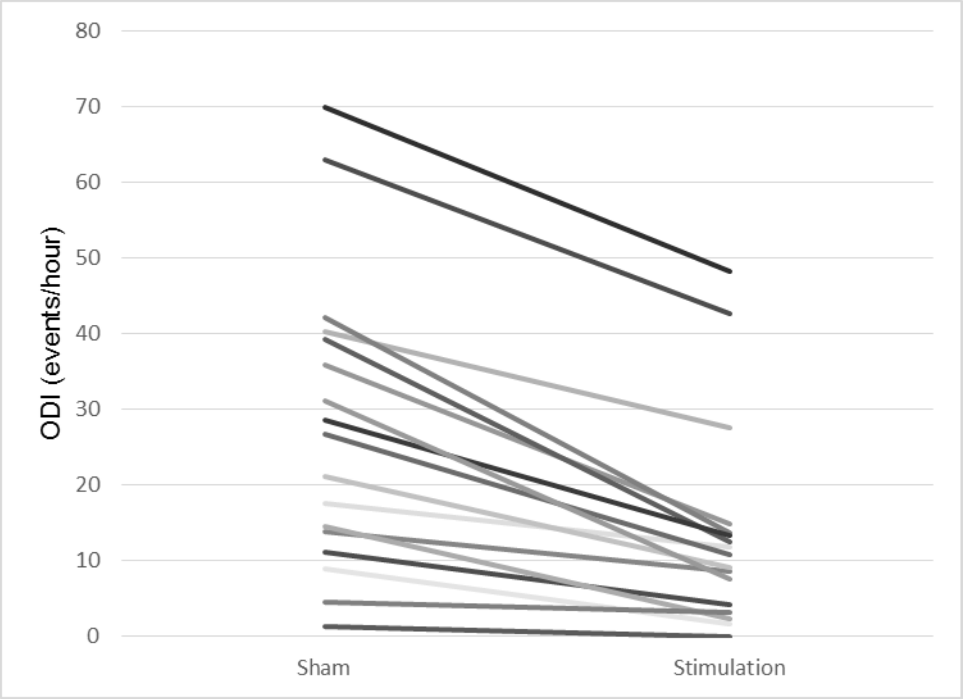

Figure E4: Individual responses to intervention in the 4%ODI for the responder group (n=17).

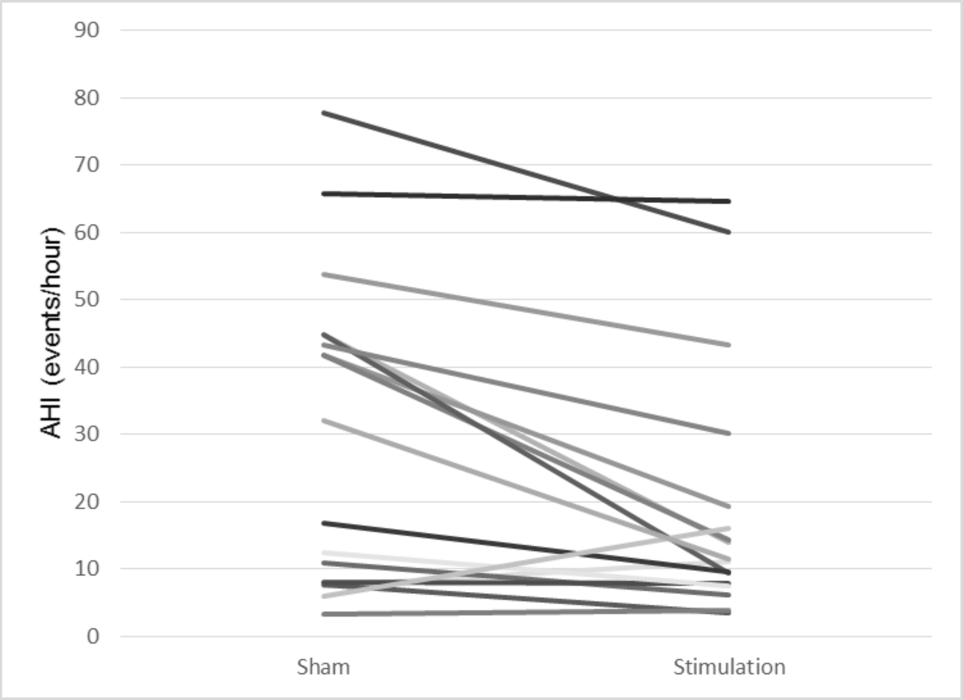

Figure E4: Individual responses to intervention in the AHI for the responder group (n=17).

Multiple Linear Regression Analysis

Model Summary

Model	R	R Square	Adjusted R Square	Std. Error of the Estimate	Change Statistics				
					R Square Change	F Change	df1	df2	Sig. F Change
1	.348[a]	.121	.095	12.54314	.121	4.695	1	34	.037

a. Predictors: (Constant), baseline 4%ODI

Coefficients[a]

Model		Unstandardized Coefficients		Standardized Coefficients	t	Sig.	95.0% Confidence Interval for B	
		B	Std. Error	Beta			Lower Bound	Upper Bound
1	(Constant)	.085	3.695		.023	.982	-7.424	7.594
	Baseline 4%ODI	.192	.089	.348	2.167	.037	.012	.372

a. Dependent Variable: 4%ODI response

Excluded Variables[a]

Model		Beta In	t	Sig.	Partial Correlation	Collinearity Statistics
						Tolerance
1	age	.031[b]	.190	.851	.033	.991
	gender	.060[b]	.361	.720	.063	.968

a. Dependent Variable: 4%ODI response

b. Predictors in the Model: (Constant), baseline 4%ODI

References

E1. Schulz KF, Altman DG, Moher D, CONSORT Group. CONSORT 2010 statement: updated guidelines for reporting parallel group randomised trials. *BMJ* 2010;340:c332.

E2. Miller MR, Hankinson J, Brusasco V, et al, ATS/ERS Task Force. Standardisation of spirometry. *Eur Respir J* 2005;26:319–338.

E3. Guidelines for the measurement of respiratory function. Recommendations of the British Thoracic Society and the Association of Respiratory Technicians and Physiologists. *Respir Med* 1994;88:165–194.

E4. Johns MW. A new method for measuring daytime sleepiness: the Epworth sleepiness scale. *Sleep* 1991;14:540–545.

E5. Kushida CA, Littner MR, Morgenthaler T, et al. Practice parameters for the indications for polysomnography and related procedures: an update for 2005. *Sleep* 2005;28:499–521.

E6. Berry RB, Budhiraja R, Gottlieb DJ, et al, American Academy of Sleep Medicine. Rules for scoring respiratory events in sleep: update of the 2007 AASM Manual for the Scoring of Sleep and Associated Events. Deliberations of the Sleep Apnoea Definitions Task Force of the American Academy of Sleep Medicine. *J Clin Sleep Med* 2012;8:597–619.

E7. Hoddes E, Zarcone V, Smythe H, et al. Quantification of sleepiness: a new approach. *Psychophysiology* 1973;10:431–436.

E8. Steier J, Seymour J, Rafferty GF, et al. Continuous transcutaneous submental electrical stimulation in obstructive sleep apnoea: a feasibility study. *Chest* 2011;140:998–1007.
